# *Amblyomma sculptum* Salivary PGE_2_ Modulates the Dendritic Cell-*Rickettsia rickettsii* Interactions *in vitro* and *in vivo*

**DOI:** 10.3389/fimmu.2019.00118

**Published:** 2019-02-04

**Authors:** Eliane Esteves, Bruna Bizzarro, Francisco Borges Costa, Alejandro Ramírez-Hernández, Ana Paula Ferranti Peti, Allan Henrique Depieri Cataneo, Pryscilla Fanini Wowk, Rodolfo Pessato Timóteo, Marcelo Bahia Labruna, Pedro Ismael Silva Junior, Célio Lopes Silva, Lúcia Helena Faccioli, Andréa Cristina Fogaça, Carlos Arterio Sorgi, Anderson Sá-Nunes

**Affiliations:** ^1^Department of Immunology, Institute of Biomedical Sciences, University of São Paulo, São Paulo, Brazil; ^2^Department of Preventive Veterinary Medicine and Animal Health, School of Veterinary Medicine and Animal Science, University of São Paulo, São Paulo, Brazil; ^3^Department of Clinical Analysis, Toxicology and Food Science, School of Pharmaceutical Sciences of Ribeirao Preto, University of São Paulo, Ribeirao Preto, Brazil; ^4^Laboratory of Molecular Virology, Carlos Chagas Institute, Fundação Oswaldo Cruz, Curitiba, Brazil; ^5^Institute of Natural and Biological Sciences, Federal University of Triângulo Mineiro, Uberaba, Brazil; ^6^Special Laboratory of Applied Toxinology, Butantan Institute, São Paulo, Brazil; ^7^Department of Biochemistry and Immunology, School of Medicine of Ribeirao Preto, University of São Paulo, Ribeirao Preto, Brazil; ^8^Department de Parasitology, Institute of Biomedical Sciences, University of São Paulo, São Paulo, Brazil; ^9^National Institute of Science and Technology in Molecular Entomology, National Council of Scientific and Technological Development (INCT-EM/CNPq), Rio de Janeiro, Brazil

**Keywords:** *Amblyomma sculptum*, tick saliva, PGE_2_, dendritic cells, *Rickettsia rickettsii*, immunomodulation

## Abstract

*Amblyomma sculptum* is an important vector of *Rickettsia rickettsii*, causative agent of Rocky Mountain spotted fever and the most lethal tick-borne pathogen affecting humans. To feed on the vertebrate host's blood, *A. sculptum* secretes a salivary mixture, which may interact with skin resident dendritic cells (DCs) and modulate their function. The present work was aimed at depicting the *A. sculptum* saliva-host DC network and the biochemical nature of the immunomodulatory component(s) involved in this interface. *A. sculptum* saliva inhibits the production of inflammatory cytokines by murine DCs stimulated with LPS. The fractionation of the low molecular weight salivary content by reversed-phase chromatography revealed active fractions eluting from 49 to 55% of the acetonitrile gradient. Previous studies suggested that this pattern of elution matches with that observed for prostaglandin E_2_ (PGE_2_) and the molecular identity of this lipid mediator was unambiguously confirmed by a new high-resolution mass spectrometry methodology. A productive infection of murine DCs by *R. rickettsii* was demonstrated for the first time leading to proinflammatory cytokine production that was inhibited by both *A. sculptum* saliva and PGE_2_, a result also achieved with human DCs. The adoptive transfer of murine DCs incubated with *R. rickettsii* followed by treatment with *A. sculptum* saliva or PGE_2_ did not change the cytokine profile associated to cellular recall responses while IgG2a-specific antibodies were decreased in the serum of these mice. Together, these findings emphasize the role of PGE_2_ as a universal immunomodulator of tick saliva. In addition, it contributes to new approaches to explore *R. rickettsii*-DC interactions both *in vitro* and *in vivo*.

## Introduction

Ticks are hematophagous ectoparasites that transmit a broad range of pathogens, causing several diseases in humans and other animals (1). The physiology of the feeding process is different between the Argasidae (soft ticks) and the Ixodidae (hard ticks) families. While blood meal acquisition by Argasidae ticks is usually fast, taking from minutes to a few hours, Ixodidae ticks remain attached from days to weeks before dropping off the host (1). Blood feeding by ticks is a dynamic event and the salivary secretion has a pivotal role during the process, providing the control of the osmotic balance (2) and presenting a plethora of molecules with antihemostatic, anti-inflammatory, and immunomodulatory properties (3–5). As a consequence of modulating host hemostasis and immune responses, tick saliva may facilitate both transmission and acquisition of pathogens (3), which might correlate with the wide range of pathogens they transmit, including bacteria, viruses, protozoa and helminths (6–8). The phenomenon of pathogen infectivity enhancement caused by tick saliva was called saliva-activated transmission (SAT), a term originally coined to describe the promotion of Thogoto virus transmission by the salivary gland extract (SGE) of *Rhipicephalus appendiculatus* (9). Henceforward, several studies reported the SAT for many other viruses and bacteria, revealing the role of tick saliva in the increased infectivity of microorganisms in the blood-feeding context (3).

The most lethal among tick-borne diseases affecting humans is Rocky Mountain spotted fever, also known as Brazilian spotted fever, caused by *Rickettsia rickettsii* (10–14). In Brazil, the southeast region is the most affected (specifically the state of Sao Paulo) which contains the majority of the cases and the highest case-fatality rate (55%) (12, 14). In the Brazilian territory, the confirmed vectors of Rocky Mountain/Brazilian spotted fever, are *Amblyomma sculptum* [formely *Amblyomma cajennense*] (15) and *Amblyomma aureolatum* (12, 16).

During feeding, ticks insert their mouthparts into the skin of the host causing local tissue damage. Skin resident dendritic cells (DCs) work as sensors of the environment by interacting with commensal microorganisms and inflammatory stimuli (17–19). As a result, DCs promote tissue homeostasis (20), tolerance (21–23), and activation of T cell responses during infectious processes (24). The dynamics of tick saliva-DC interactions was first approached by studies showing that Langerhans cells—a major DC population from the epidermis—trap antigens from tick salivary glands (25, 26) and present them to lymphocytes in draining lymph nodes (27). These cells are also associated with tick resistance (28) and were found surrounding tick mouthparts in secondary infestations (29). More recently, a number of studies demonstrated that tick saliva affects the biology of DCs, typically inhibiting their differentiation, maturation, and function (30–35). Indeed, several molecules responsible for DC immunomodulation have been identified and characterized in salivary preparations of *Ixodes scapularis* (31, 36–39), *Rhipicephalus sanguineus* sensu lato (40), *R. appendiculatus* (41) and *Ornithodoros moubata* (42, 43). However, the identity of the putative molecule(s) present in *A. sculptum* saliva involved in DC modulation is elusive to date.

In the present work, we demonstrated the immunomodulatory effect of *A. sculptum* saliva on cytokine production by LPS-stimulated DCs. By employing bioassay-guided fractionation methods associated to a recently developed high-resolution mass spectrometry technique for target lipids, we ultimately characterized PGE_2_ as the molecule responsible for this biological activity in *A. sculptum* saliva. In addition, we showed for the first time that *A. sculptum* saliva and PGE_2_ inhibit the production of some proinflammatory cytokines induced by *R. rickettsii* in murine and human DCs. Our results also revealed that both saliva and PGE_2_ modulate adoptively transferred DCs to induce changes in humoral immune responses to *R. rickettsii in vivo*.

## Materials and Methods

### Ethics Statement

All procedures involving vertebrate animals were carried out in accordance with the Brazilian National Law number 11,794 and approved by the Institutional Animal Care and Use Committee from the University of Sao Paulo (protocol numbers 1423/2008, 128/2011 and 55/2015). All procedures involving humans were carried out in accordance with the recommendations of the Brazilian National Health Council, National Research Ethics Commission. The protocol was approved by the Human Research Ethics Committee from Fiocruz (CAAE: 60643816.6.0000.5248). All subjects gave written informed consent in accordance with the Declaration of Helsinki.

### Animals

C3H/HePas female mice (*Mus musculus*), 6–12-week-old, were bred and maintained at the Isogenic Breeding Unit from the Department of Immunology, Institute of Biomedical Sciences, University of Sao Paulo, Sao Paulo, Brazil. New Zealand male rabbits (*Oryctolagus cuniculus*), 10–12-week-old, were purchased from ANILAB (Paulínia, SP, Brazil) and maintained at the Animal Facility from the Department of Preventive Veterinary Medicine and Animal Health, School of Veterinary Medicine and Animal Science, University of Sao Paulo, Sao Paulo, Brazil.

### Ticks and Saliva Collection

*Amblyomma sculptum* ticks were obtained either from a laboratory colony started with adult ticks collected at Pedreira municipality, Sao Paulo State, Brazil or from the field, collected at Uberaba municipality, Minas Gerais State, Brazil. Larvae, nymphs, and adults were fed on rabbits as previously described (44). Off-host phases were held in an incubator at 25°C and 95% relative humidity. Unless otherwise indicated, adult *A. sculptum* females were removed from the vertebrate hosts after 7–9 days of attachment, washed in sterile phosphate-buffered saline (PBS), and salivation was induced by injection of pilocarpine (50 mg/mL in 0.7 M NaCl) or dopamine (0.2% in PBS) into the tick hemocoel using a 12.7 × 0.33 mm BD Ultra-Fine™ needle (Becton, Dickinson and Company, Franklin Lakes, NJ, United States) as previously described (45). The saliva was harvested every 10–15 min using a micropipette and transferred to a polypropylene tube kept on ice. Samples were stored at−80°C until use. The concentration of pilocarpine in the saliva samples was determined by mass spectrometry (Accela TSQ Quantum Max) at the Research Center Facility (CEFAP), Institute of Biomedical Sciences, University of Sao Paulo.

### *R. rickettsii* Culture

*R. rickettsii*, Taiaçu strain (46) were grown until the 5th passage in VERO cells (ATCC™ CCL-81^TM^, Manassas, VA, United States). When 100% of VERO cells were infected, confirmed by Giménez stain (47), cells were collected, centrifuged at 4,500 *g* for 10 min and resuspended in sucrose-phosphate-glutamate buffer (48). Aliquots of 200 μL were transferred to cryovials and maintained in liquid nitrogen until use. For the experiments, the cryovials were immersed in water bath at 37°C until complete thawing followed by incubation in liquid nitrogen for 5 min, for cell disruption and bacteria release.

### Fractionation of *A. sculptum* Saliva

Fifty microliters of *A. sculptum* saliva, collected after 7–9 days of host attachment, were diluted in 450 μL of PBS, and filtrated through a 3-kDa molecular weight cutoff microfilter (Vivaspin 500, Sartorius Biolab Products, Goettingen, NI, Germany), separating the saliva into a low molecular weight (LMW; < 3 kDa) and a high molecular weight (HMW; >3 kDa) fractions. The HMW fraction was diluted again in PBS and filtrated a second time. The resulting HMW and LMW fractions were pooled, sterilized through a 0.22 μm membrane (Millipore Corporation, Billerica, MA, United States), and used in DC cultures described later.

Two hundred microliters of the LMW fraction of *A. sculptum* saliva were applied onto analytical reversed phase C_18_ column (Shim-pack Shimadzu VP-ODS, size 250 mm × 4.6 mm, 5 μm), coupled to a ultra fast liquid chromatography (UFLC) system (LC-20AT Prominence, Shimadzu, Japan) previously equilibrated with 2% acetonitrile (ACN) in acidified water (0.05% trifluoroacetic acid). Salivary molecules were eluted with a linear gradient of 2–60% ACN in acidified water over 60 min, at a flow rate of 1 mL/min. Fractions were individually used in DC cultures described later.

### Murine Bone Marrow-Derived DC Cultures

Bone marrow cells from C3H/HePas mice were collected from the femur and adjusted to 3 × 10^6^ cells/mL in complete medium [RPMI 1640 medium supplemented with 10% heat-inactivated fetal bovine serum (FBS), 2 mM L-glutamine, 100 U/mL penicillin, 100 μg/mL streptomycin, 2.5 × 10^5^ M 2-mercaptoethanol (all from GIBCO™, Grand Island, NE, United States)] in the presence of 20 ng/mL of murine GM-CSF (Biolegend, San Diego, CA, United States) to induce DC differentiation, as previously described (49). After 4 days, half of the medium was collected and replaced with fresh complete medium containing 40 ng/mL of GM-CSF. On the 7th day of culture, non-adherent cells were collected, washed, resuspended at 10^6^ cells/mL in complete medium and distributed into 96-well plates at 10^5^ cells/well.

For different experiments, cells were preincubated for 1 h with *A. sculptum* saliva collected after 7–9 days of host attachment (1:50, 1:100, 1:500, and 1:1,000 dilution), pilocarpine (0.5, 0.1, 0.05, 0.01 mM), LMW or HMW saliva fractions (equivalent to a 1:50 dilution of the whole saliva), or 100 nM (final concentration) of commercial PGE_2_ (Cayman Chemical, Ann Arbor, MI, United States), followed by incubation with 200 ng/mL of ultrapure lipopolysaccharide (LPS; InvivoGen, San Diego, CA, United States) or 10^6^ *R. rickettsii* at 37°C under 5% CO_2_.

In another set of experiments, a cell suspension containing 10^6^ cells/mL was prepared, distributed into 96-well plates at 10^5^ cells/well, and preincubated for 1 h with UFLC fractions derived from *A. sculptum* saliva (equivalent to a 1:50 dilution of the whole saliva) followed by stimulation with LPS (200 ng/mL) at 37°C under 5% CO_2_.

Cytokines were evaluated in cell-free supernatants collected after 6 h (for TNF-α) or 18 h (for IL-6, IL-12p40 and IL-12p70) by enzyme-linked immunosorbent assay (ELISA) using BD OptEIA™ ELISA Sets (BD Biosciences, San Diego, CA, United States) according to manufacturer's instructions.

For flow cytometry analysis, DCs were preincubated for 1 h with medium only, *A. sculptum* saliva (1:50 dilution) or PGE_2_ (100 nM-final concentration), followed by incubation with *R. rickettsii* for 18 h, as described above. DCs were collected, washed twice in culture medium to remove unbound bacteria, suspended in PBS containing 1% heat-inactivated FBS and stained with fluorochrome-conjugated antibodies against murine CD11c, I-A/I-E (MHC class II), CD40, CD80, and CD86 (BD Biosciences), acquired in a FACSCanto II (BD Biosciences) and analyzed by FlowJo software, version 10.0.7 (Tree Star, Ashland, OR, United States).

### Human Monocyte-Derived DC Cultures

Peripheral blood samples were obtained by venipuncture from three healthy donors. Peripheral blood mononuclear cells (PBMC) were obtained by density gradient separation with Ficoll-Paque PLUS, density 1.077 g/mL (GE Healthcare, Chicago, IL, United States). CD14^+^ cells were purified by positive selection with anti-human CD14 microbeads (Miltenyi Biotec, Auburn, CA, United States) in accordance with the manufacturer's recommendations. For DC differentiation, CD14^+^ cells were maintained in complete medium containing 25 ng/mL of human IL-4 and 12.5 ng/mL of human GM-CSF (both from PeproTech, Rocky Hill, NJ, United States) and incubated at 37°C under 5% CO_2_. After 3 days, fresh medium containing IL-4 and GM-CSF was added to the culture. On the 7th day of culture, non-adherent cells were collected, washed and suspended at 10^6^ cells/mL in complete medium. DCs were distributed into 96-well plates at 10^5^ cells/well and preincubated for 1 h with medium only, *A. sculptum* saliva (1:50 dilution) or commercial PGE_2_ (100 nM-final concentration), followed by incubation with 10^6^ *R. rickettsii* at 37°C under 5% CO_2_. After 24 h, the cell-free supernatants were collected for TNF-α, IL-6, IL-12p70, IL-8, and IL-1β determination by cytometric bead array (CBA-BD Biosciences) according to manufacturer's instructions.

For flow cytometry analysis, cells were recovered with 100 μL of blocking buffer (PBS supplemented with 5% FBS and 1% AB human serum) and incubated for 20 min at room temperature. After centrifugation, cells were stained with fluorochrome-conjugated antibodies against human CD11c, HLA-DR, CD40, CD80, and CD86 (BD Biosciences), acquired in a FACSCanto II (BD Biosciences) and analyzed by FlowJo software, version 10.0.7 (Tree Star).

### PGE_2_ Determination

PGE_2_ concentration in *A. sculptum* saliva collected after 3, 5, 7, 9, and 11 days of host attachment or in saliva fractions between 49 and 55% ACN was determined by a PGE_2_ EIA Kit (Cayman) following the manufacturer's instructions.

### Target Lipids Mass Spectrometry

Eicosanoids from saliva and saliva fractions were purified by ACN extraction (liquid/liquid). The solvent was added to the sample (sample:ACN-1:10 *v/v*) and maintained overnight at −80°C. After, the samples were centrifuged, the organic phase was obtained, dried and resuspended in 50 μL of methanol:water (7:3 *v/v)*. Aliquots of each extracted sample were injected into an LC-MS/MS system equipped with an ESI (electrospray source ionization) operated in negative mode on TripleTOF 5600® mass spectrometer (Sciex, Redwood City, CA, United States). Compounds separation employed a binary gradient with phase A (water:acetonitrile-7:3 *v/v*; pH 5.8) and phase B (acetonitrile:isopropanol-7:3 *v/v)* at a flow of 0.6 mL/min in an Ascentis Express C_18_ column (Supelco, St. Louis, MO, United States). The characterization of PGE_2_ was performed by the high-resolution multiple-reaction monitoring (MRM^HR^) method with specific *m/z* transitions for enabled proper alignment of the detected fragment ions with their respective precursor ions for accurate identification in high-resolution and matching with standards analytes (Cayman) as previously described (50).

### Transmission Electron Microscopy

Murine DCs were distributed into 24-well plates at 10^6^ cells/well and incubated for 18 h at 37°C under 5% of CO_2_ with 10^7^ *R. rickettsii*, Taiaçu strain (46). Cells were collected and fixed at 4°C with 2% glutaraldehyde and 2% paraformaldehyde in 0.1 M cacodylate buffer, pH 7.4, post-fixed in 1% osmium tetroxide (OsO_4_) and embedded in Spurr resin. Ultrathin sections of 70 nm were gathered onto copper grids and stained with 2% uranyl acetate for 1 h, then washed in distilled water and stained in 0.5% lead citrate (51). Ultrastructure was examined in a TECNAI G20 200 KV electron microscope (FEI Company, Eindhoven, Netherlands).

### Adoptive Transfer of DCs Incubated With *R. rickettsii* to Mice

Murine DCs were distributed into 24-well plates at 10^6^ cells/well and preincubated with medium only (control), *A. sculptum* saliva (1:50 dilution) or PGE_2_ (100 nM-final concentration) for 1 h, followed by incubation with 10^7^ *R. rickettsii* for 18 h at 37°C under 5% CO_2_. DCs of each group were collected and washed twice in culture medium to remove unbound bacteria and resuspended in PBS. Mice were injected subcutaneously with 10^6^ DCs of each group in a 100 μL volume. During the first 5 days after DC transfer, animals of each group received a subcutaneous treatment (100 μL at the same site of DC inoculation) as follows: PBS (group “PBS”); *A. sculptum* saliva 1:50 dilution (group “Saliva”); and 100 nM PGE_2_ (group “PGE_2_”). After 30 days, mice were euthanized and the blood (serum separation) and spleen (lymphoproliferation and qPCR) were collected.

### Lymphocyte Proliferation and Cytokine Production

Spleens from mice adoptively transferred with DCs were aseptically dissected after euthanasia and spleen cells were separated using a 40 μm cell strainer (Corning Cell Strainer, Corning Incorporated, Durham, NC, United States). A cell suspension was prepared and transferred to 96-well plates at 10^5^ cells/well followed by stimulation with concanavalin A (Con A-0.5 μg/mL final concentration; Sigma-Aldrich, St Louis, MO, United States) or heat-killed *R. rickettsii* (HKRr−2 × 10^5^ bacteria/well) and incubated at 37°C under 5% CO_2_. After 48 h, 25 μL of 0.01% resazurin (prepared in complete medium) were added to culture cells and after additional 24 h, the culture absorbance at 570 and 600 nm was determined and used to indirectly evaluate cell proliferation as previously described (31, 36, 49).

In another set of experiments, spleen cells were distributed into 24-well plates at 2.5 × 10^6^ cells/well followed by stimulation with 5 × 10^6^ cells of HKRr/well. After 72 h of incubation at 37°C under 5% CO_2_, the concentration of IFN-γ and IL-4 was evaluated in cell-free supernatants according to manufacturer's instructions (BD OptEIA™ ELISA Set-BD Biosciences).

### IgG1 and IgG2a Determination

Specific IgG1 and IgG2a against *R. rickettsii* antigens were measured in the serum of mice receiving DC adoptive transfer under different conditions by an in-house ELISA assay. Briefly, 96-well plates were coated with HKRr (equivalent to 10^6^ bacteria/well) diluted in 0.1 M sodium carbonate buffer (pH 9.5) and maintained overnight at 4°C, followed by blocking with PBS containing 1% heat-inactivated FBS for 1 h at room temperature. Serum samples (1:100 dilution) were added and incubated for 2 h. Peroxidase-labeled anti-IgG1 (Invitrogen, Waltham, MA, United States) and -IgG2a (BD Biosciences) detection antibodies (1:1,000 dilution) were added for 1 h and the reaction was developed by addition of the TMB substrate reagent set (BD Biosciences). The reaction was stopped by addition of phosphoric acid [(H_3_PO_4_)1 M] and the absorbance at 450 nm was determined and used to qualitatively estimate the amount of each antibody. The blank for each reaction consisted of the same dilutions of serum from mice injected with DCs only.

### Statistical Analysis

For the comparison of the experimental groups, analysis of variance (ANOVA) followed by Tukey as a post-test was used. A *p* ≤ 0.05 was considered statistically significant.

## Results

### *A. sculptum* Saliva Inhibits LPS-Induced Proinflammatory Cytokine Production

**Figure 1:**
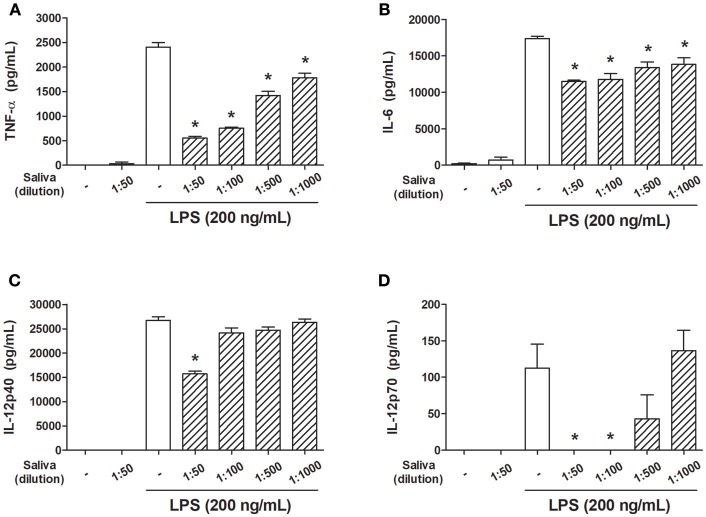
*A. sculptum* saliva inhibits the production of inflammatory cytokines by LPS-stimulated murine DCs. Bone marrow cells from C3H/HePas mice were differentiated into DCs, seeded at 10^5^ cells/well in 96-well plates and preincubated for 1 h with medium only or with different dilutions of *A. sculptum* saliva (indicated in the figure) collected by pilocarpine stimulation from female ticks fed on host for 7–9 days. Cells were stimulated with ultrapure LPS (200 ng/mL) during 6 h for TNF-α **(A)** or 18 h for IL-6, IL-12p40, and IL-12p70 **(B–D, respectively)** determination in culture supernatants by ELISA. Results are expressed as the mean ± SEM. ^*^p ≤ 0.05 *vs*. “LPS without saliva” group.

In order to evaluate the effects of *A. sculptum* saliva on parameters associated with DC maturation, cells were preincubated with different dilutions of saliva (induced by pilocarpine) and stimulated with LPS. A significant inhibition of LPS-induced TNF-α and IL-6 production by DCs was achieved at all saliva dilutions employed (Figures 1A,B) whereas IL-12p40 production was significantly decreased only at 1:50 dilution (Figure 1C), and IL-12p70 production was decreased at 1:50 and 1:100 dilution (Figure 1D).

In order to determine whether the presence of pilocarpine in the saliva samples could influence the response of DCs, the concentration of the salivary agonist was determined by mass spectrometry. The quantification of five independent samples indicated a pilocarpine salivary concentration of 1,555.04 ± 513.01 μg/mL (~7.5 mM). In the presence of 0.5 mM pilocarpine (more than 3-fold the equivalent of saliva at 1:50 dilution, which is expected to be around 0.15 mM) we observed a small inhibition of TNF-α production by DCs but not of IL-6, IL-12p40, or IL-12p70. At lower concentrations (0.1, 0.05 and 0.01 mM) no changes in the production of these cytokines were observed in the presence of the agonist (Supplementary Figure S1). In addition, experiments performed in the presence of saliva induced by dopamine revealed a similar inhibitory pattern of all cytokines evaluated when compared with pilocarpine-induced saliva (Supplementary Figure S2). Therefore, both salivary preparations were considered equivalent in terms of their biological activity on dendritic cells, irrespective of the agonist used for salivation.

### PGE_2_ Is the Major DC Inhibitor of *A. sculptum* Saliva

**Figure 2:**
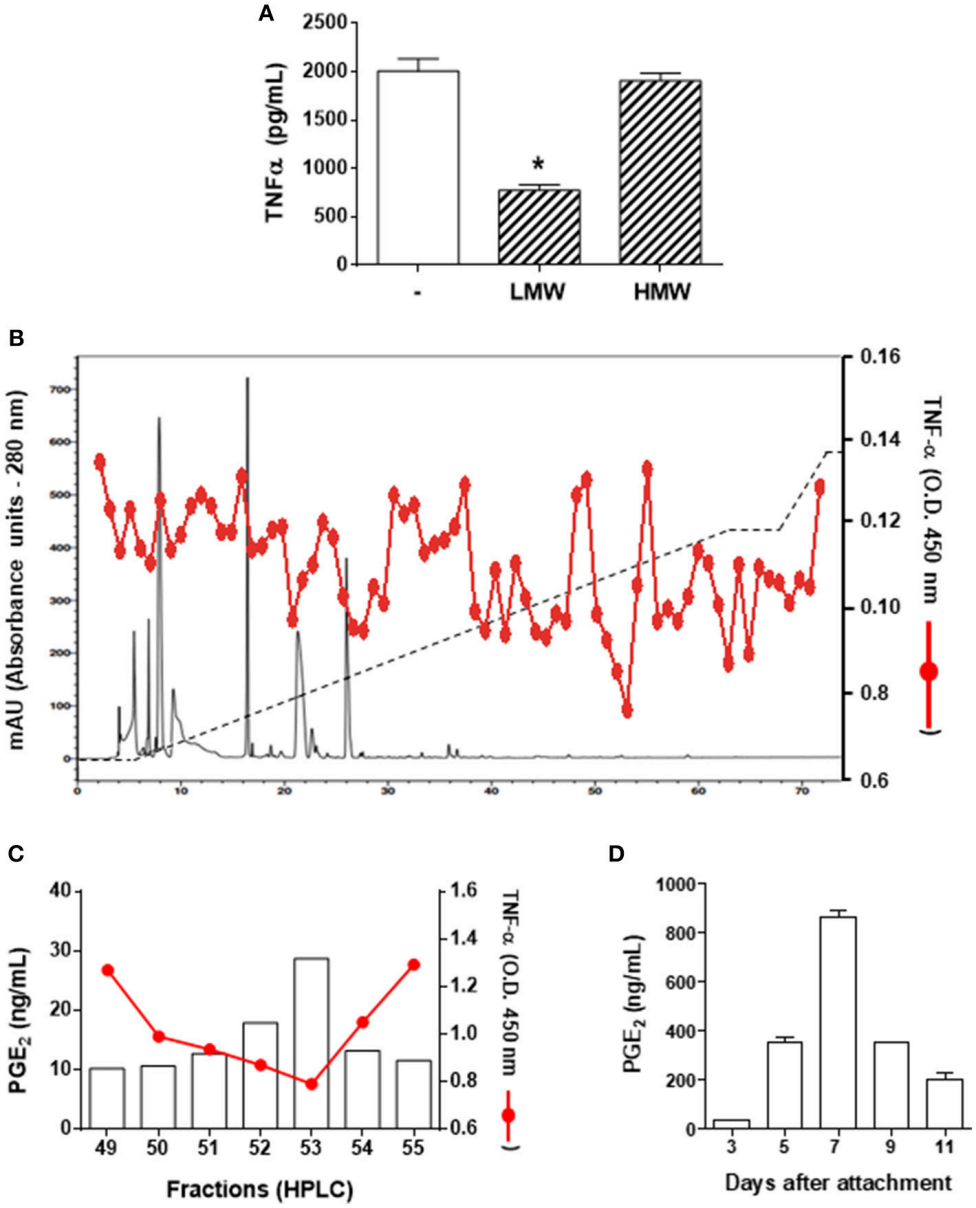
PGE_2_ is the DC modulatory molecule of *A. sculptum* saliva. Bone marrow cells from C3H/HePas mice were differentiated into DCs, seeded at 10^5^ cells/well in 96-well plates and preincubated for 1 h with medium only, with a low molecular weight (LMW) or with a high molecular weight (HMW) fractions of *A. sculptum* saliva collected by pilocarpine stimulation from female ticks fed on host for 7–9 days. Cells were stimulated with ultrapure LPS (200 ng/mL) and after 6 h, the TNF-α production was evaluated in culture supernatants by ELISA **(A)**. The LMW fraction was fractionated by UFLC (solid black line), eluted with a linear gradient of ACN (2–60% -dashed black line). Each fraction was tested for LPS-induced TNF-α production by DCs (solid red line) **(B)**. PGE_2_ concentration was determined in the fractions presenting stronger inhibition of TNF-α (eluted between 49 to 55% of ACN) by ELISA **(C)**. PGE_2_ concentration was determined in *A. sculptum* saliva collected by pilocarpine stimulation from female ticks fed on host for 3, 5, 7, 9, and 11 days **(D)**. ^*^p ≤ 0.05 *vs*. “LPS without fractions” group.

To characterize the salivary molecule(s) associated to DC immunomodulation, *A. sculptum* saliva components were initially separated into a LMW fraction (< 3 kDa) and a HMW fraction (> 3 kDa) by microfiltration. DCs were maintained in medium or preincubated with each fraction followed by LPS stimulation. The production of TNF-α was evaluated as a readout parameter of cell activation, since it was the cytokine most inhibited in the presence of saliva (Figure 1A). As observed in the Figure 2A, the LMW fraction significantly inhibited TNF-α production while the HMW fraction did not affect the production of the cytokine. Of note, the LMW fraction also inhibited the production of IL-6, IL-12p40, and IL-12p70 (data not shown).

*A. sculptum* saliva components were fractionated by reversed phase UFLC and the activity of each fraction was screened on LPS-stimulated DCs, using the TNF-α production as a readout assay. The stronger inhibition of TNF-α production occurred in the presence of fractions eluted between 49 and 55% of ACN (Figure 2B). Previous fractionation approaches of *I. scapularis* and *R. sanguineus* saliva employing similar chromatographic conditions identified PGE_2_ as the DC immunomodulatory molecule in fractions eluted at comparable ACN concentrations (31, 40). Therefore, the presence of PGE_2_ was evaluated in the fractions presenting TNF-α inhibitory using a commercial PGE_2_ ELISA kit and confirmed a direct proportional association between the potency of the activity and the concentration of this lipid mediator in the respective fraction (Figure 2C). In addition, the concentration of PGE_2_ in *A. sculptum* saliva was increased along the feeding process, reaching the peak on the 7th day after attachment to the host, and decreasing thereafter (Figure 2D).

**Figure 3:**
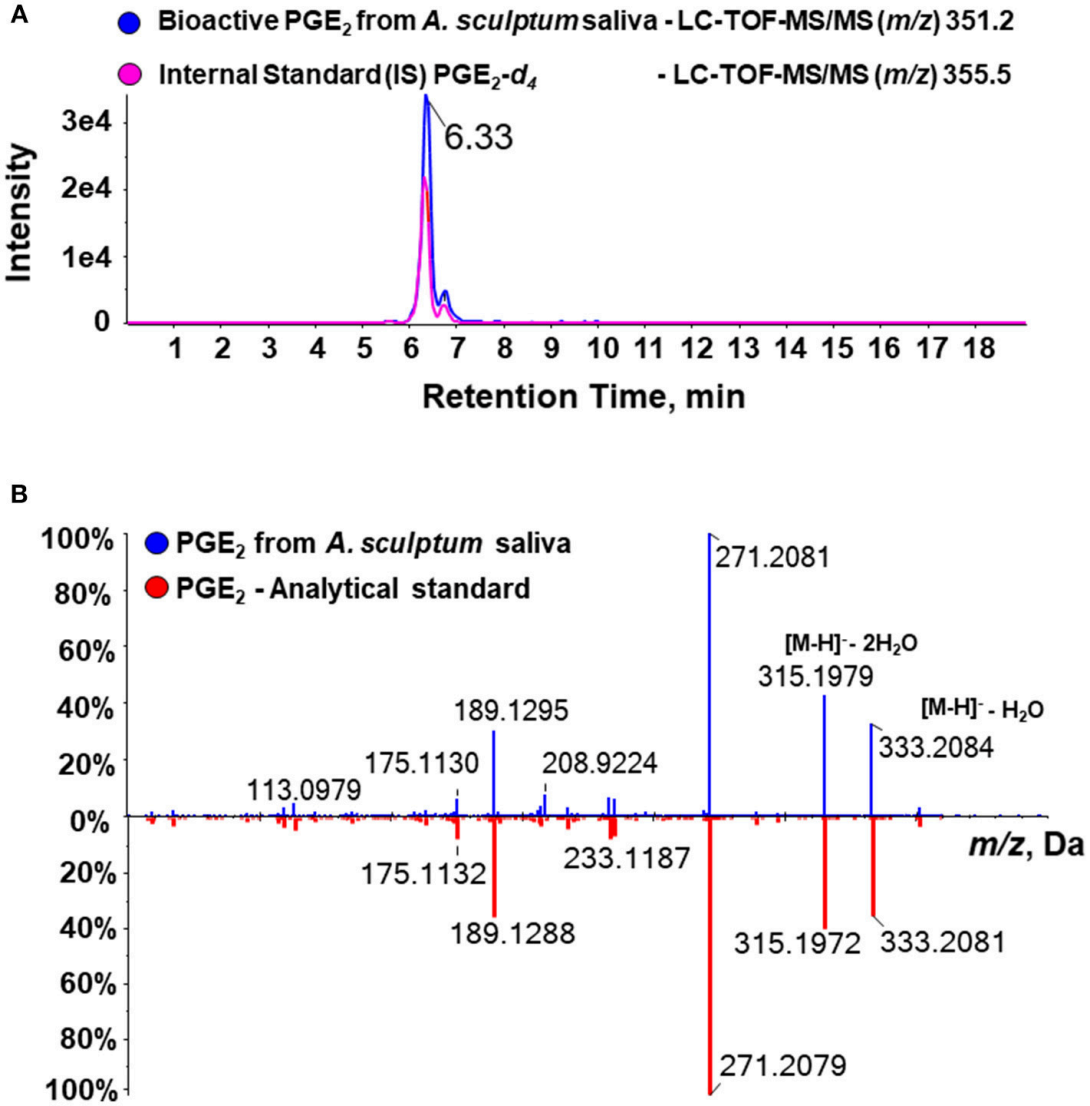
Target high-resolution sequential mass spectrometry for lipids confirms the molecular identity of PGE_2_ from *A. sculptum* saliva. Eicosanoids of *A. sculptum* saliva (collected by pilocarpine stimulation from female ticks fed on host for 7–9 days) and of saliva fractions were extracted and evaluated in an eisosanomics platform. **(A)** Chromatogram of LC-MS/MS (MRM^HR^ mode) of an internal standard (PGE_2_-*d*_4_ – pink line) and the target bioactive PGE_2_ (blue line) showing the same retention time. **(B)** Comparative fragmental spectrum in high-resolution of PGE_2_ from *A. sculptum* saliva (blue line) and a commercial PGE_2_ standard (red line) showing nearly identical *m/z* profile.

A recent study explored a new combinatory methodology involving analytical chemistry and molecular biology to describe a precise lipidomic quantitative dataset of eicosanoids in biological samples. This technique identified a cross-reaction of PGE_2_ and PGD_2_ when evaluated by commercial ELISA kits (50), thus revealing a potential artifact of previous studies that employed this assay to define the presence of PGE_2_ in tick saliva. In order to rule out any possible PGD_2_ contamination in our material (and perhaps in samples produced by similar fractionation methods), we performed a target lipidomics of our samples searching for the specific PGE_2_ signature. Bioactive fractions were analyzed in an eicosanomics platform using a MRM^HR^ method by an advanced mass spectrometry approach. The information was combined in three dimensions to give a representation of the retention time, signal intensity, and *m/z* value for the analytes and by using a bioinformatics software for qualitative review of LC-/MS data files. The data processing usually proceeds through multiple steps, including feature detection, which is conducted to identify all signals caused by true ions and to avoid the detection of false positives, thereby interpolating theoretical information from the molecular data file and experimental fragments. In our study, alignment to confirm retention-time differences between runs was conducted through a comparison of the internal standard PGE_2_-*d*_4_ chromatographic peaks. Figure 3A showed the similarity of PGE_2_-*d*_4_ retention time and bioactive PGE_2_ from *A. sculptum* salivary fraction on the analytical chromatogram. Indeed, we confirmed the real structure of PGE_2_ in *A. sculptum*-saliva by virtual match with a high-resolution fragment ion of a commercial PGE_2_ standard (Figure 3B). Also, the LC-MS/MS data were in accordance with the biological assays and the PGE_2_ quantification by an ELISA kit demonstrated in this work.

### *A. sculptum* Saliva and PGE_2_ Inhibit *R. rickettsii*-Induced Proinflammatory Cytokine Production but Not CD40, CD80, or CD86 Expression by Both Murine and Human DCS

**Figure 4:**
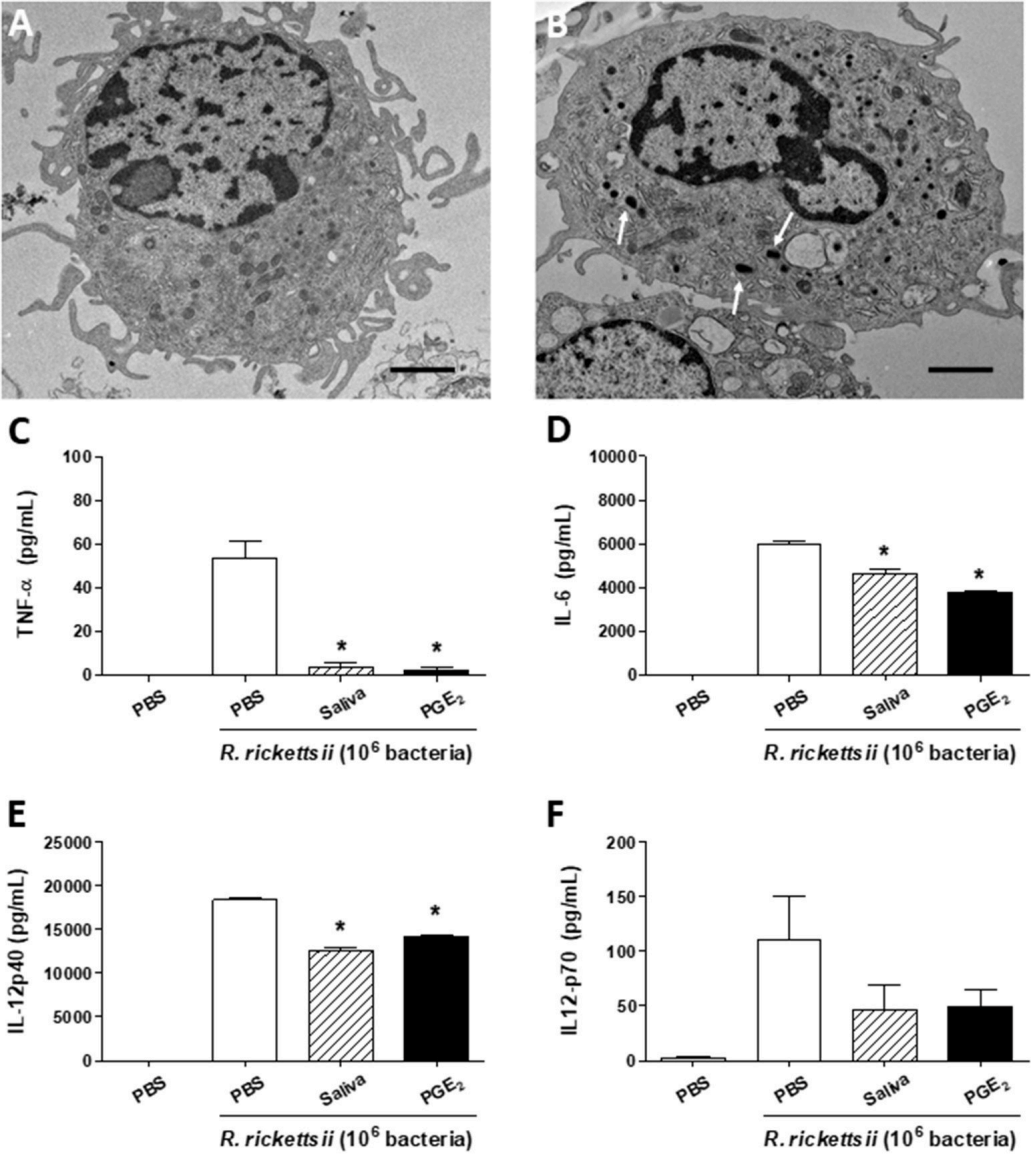
*A. sculptum* saliva and PGE_2_ downmodulate inflammatory cytokines produced by murine DCs incubated with *R. rickettsii*. Bone marrow cells from C3H/HePas mice were differentiated into DCs and used in the following assays. Transmission electron microscopy of a resting DC **(A)** and a *R. rickettsii*-infected DC **(B)**, showing the bacteria in the cytosol (white arrows). Non-adherent cells were collected, seeded at 10^5^ cells/well in 96-well plates and preincubated for 1 h with medium only, with *A. sculptum* saliva collected by pilocarpine stimulation from female ticks fed on host for 7–9 days (1:50 dilution) and PGE_2_ (100 nM). Cells were incubated with *R. rickettsii* (10^6^ bacteria) during 6 h for TNF-α **(C)** or 18 h for IL-6, IL-12p40, and IL-12p70 (**D–F**, respectively) determination in culture supernatants by ELISA. Results are expressed as the mean ± SEM. ^*^p ≤ 0.05 vs. “DC + *R. rickettsii*/PBS” group. Scale bar = 2 μM.

Recent studies reported a murine model of *R. rickettsii* infection in C3H/HeN mice (52–57). We attempted to infect C3H/HePas (a related strain) with *R. rickettsii* through a subcutaneous route, but failed to observe susceptibility (data not shown). Thus, we decided to use this murine strain to evaluate immunological parameters associated with the resistance to *R. rickettsii* infection *in vivo*, and the putative role of *A. sculptum* saliva and PGE_2_ on these parameters. By using transmission electron microscopy, we visualized the ultrastructure of a resting DC maintained in medium only (Figure 4A). The bacteria in the cytosol of the cells confirmed, for the first time, that murine DCs can be infected by *R. rickettsii in vitro* (Figure 4B, white arrows), similarly to that reported for *R. conorii* (58).

When incubated with *R. rickettsii*, murine DCs produced the proinflammatory cytokines TNF-α, IL-6 and IL-12p40 and IL-12p70 (Figures 4C–F, respectively). In the presence of *A. sculptum* saliva or PGE_2_, TNF-α, IL-6, and IL-12p40 were significantly inhibited (Figures 4C–E, respectively), similar to the response observed under LPS stimulation (Figure 1). For IL-12p70, however, the inhibition elicited by saliva and PGE_2_ did not reach statistical significance (Figure 4F).

The expression of CD40, CD80, and CD86—important maturation/activation markers of DCs and other antigen-presenting cells—was evaluated under similar conditions. DC incubation with *R. rickettsii* induced a significant expression of the three molecules when compared with cells incubated with medium only. However, the presence of *A. sculptum* saliva or PGE_2_ did not downmodulate the *R. rickettsii*-induced expression of neither of the surface marker on murine DCs (Supplementary Figure S3).

**Figure 5:**
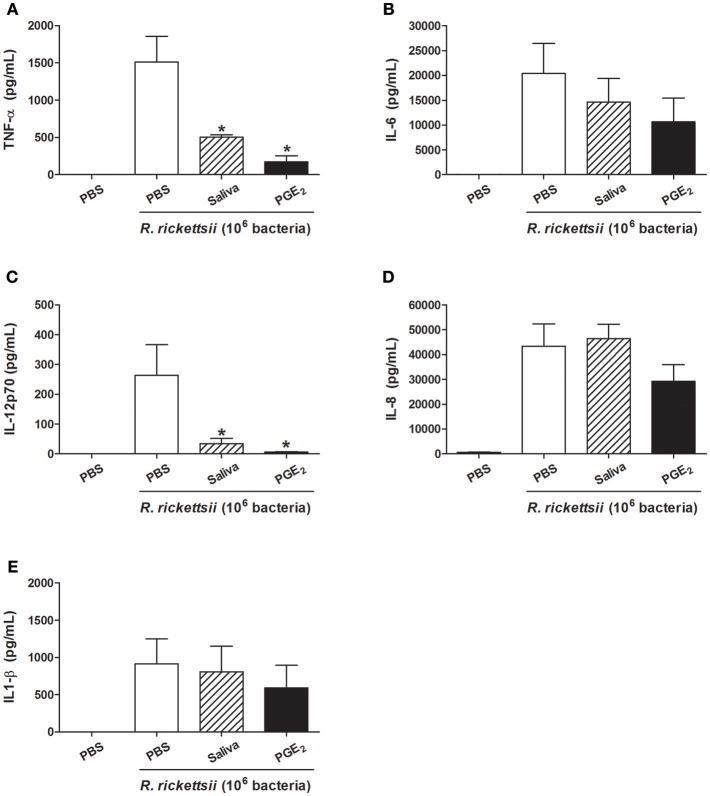
*A. sculptum* saliva and PGE_2_ downmodulate inflammatory cytokines produced by human DCs incubated with *R. rickettsii*. PBMC were differentiated into DCs, seeded at 10^5^ cells/well in 96-well plates and preincubated for 1 h with medium only, with *A. sculptum* saliva collected by dopamine stimulation from field female ticks (1:50 dilution) and PGE_2_ (100 nM). After that, cells were incubated with *R. rickettsii* (10^6^ bacteria) during 24 h for TNF-α **(A)**, IL-6 **(B)**, IL-12p70 **(C)**, IL-8 **(D)**, and IL1-β **(E)** determination in culture supernatants by CBA. Results are expressed as the mean ± SEM. ^*^p ≤ 0.05 vs. “DC + *R. rickettsii*/PBS” group.

Because *R. rickettsii* causes an infection with high case-fatality rate (55%) in humans (12, 14), we next evaluate whether the above mentioned cytokine modulation by the bacteria also occurs in human DCs. When incubated with *R. rickettsii*, human DCs produced several proinflammatory cytokines: TNF-α, IL-6, IL-12p70, IL-8, and IL-1β (Figures 5A–E, respectively). In the presence of *A. sculptum* saliva or PGE_2_, only TNF-α (Figure 5A) and IL-12p70 (Figure 5C) were significantly inhibited while IL-6 (Figure 5B), IL-8 (Figure 5D), and IL-1β (Figure 5E) presented a slight inhibition that did not reach statistical significance. The expression of CD40, CD80, and CD86 was also evaluated in the same experimental conditions. For human DCs, the incubation with *R. rickettsii*, in the presence of absence of saliva and PGE_2_, did not change the expression of CD40. A mild upregulation of CD80 was observed in the presence of *R. rickettsii*, which was partially inhibited in the presence of tick saliva. The expression of CD86 was upregulated in the presence of the bacteria, but neither saliva nor PGE_2_ diminished the expression of this surface marker in human DCs (Supplementary Figure S4).

### *A. sculptum* Saliva and PGE_2_ Change the Profile of Humoral but Not Cellular Immune Responses to *R. rickettsii*

Next, murine DC cultures maintained with medium only or treated with PGE_2_ or *A. sculptum* saliva were incubated with *R. rickettsii* and then transferred to mice. For five consecutive days the mice were treated with PBS, *A. sculptum* saliva or PGE_2_ as described in Material and Methods. After 30 days, *R. rickettsii*-specific cellular and humoral responses were evaluated.

**Figure 6:**
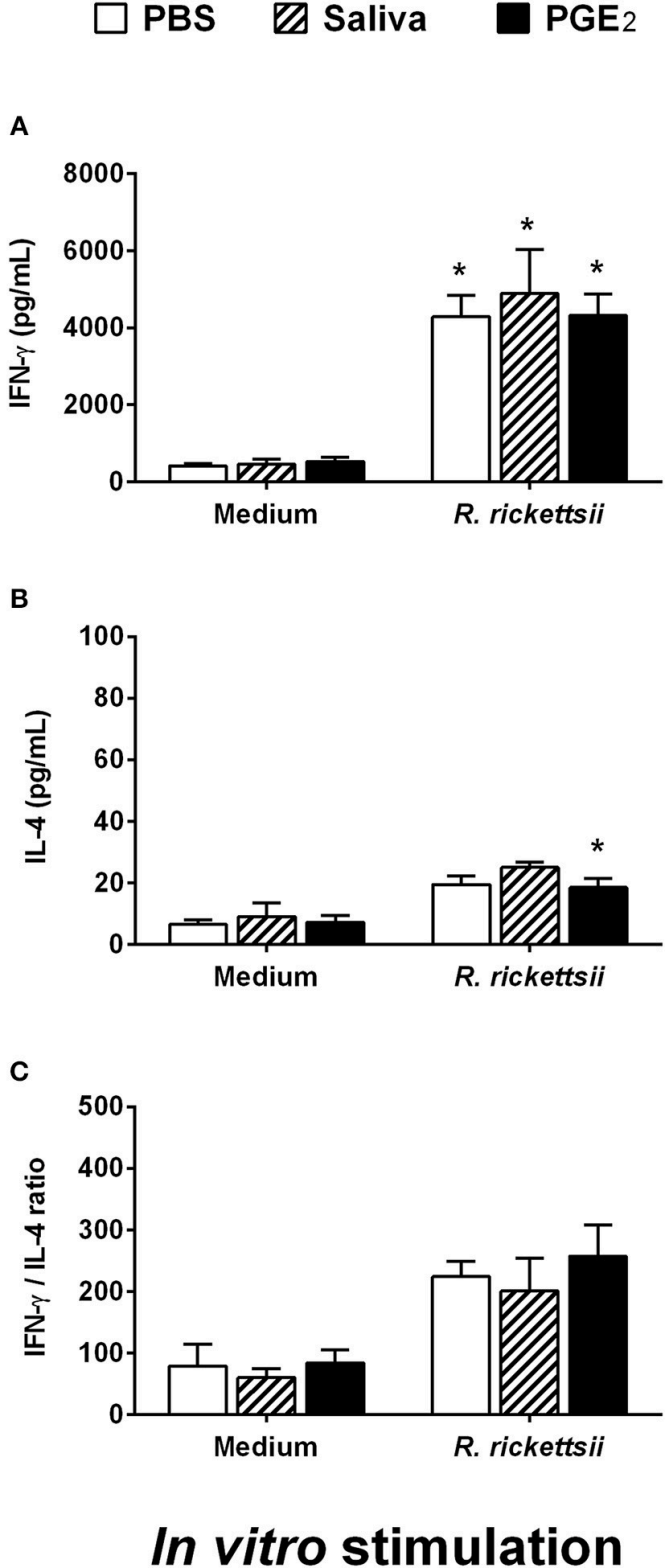
Adoptive transfer of murine DCs incubated with *R. rickettsii* and treated with *A. sculptum* saliva or PGE_2_ does not change IFN-γ or IL-4 recall response of spleen cells following antigenic restimulation *in vitro*. Bone marrow cells from C3H/HePas mice were differentiated into DCs, seeded at 10^6^ cells/well in 24-well plates and preincubated for 1 h with medium only, *A. sculptum* saliva collected by pilocarpine stimulation from female ticks fed on host for 7–9 days (1:50 dilution) or PGE_2_ (100 nM). Cells were incubated with *R. rickettsii* (10^7^ bacteria) during 18 h, collected and washed to remove unbound bacteria. Mice were injected subcutaneously with 10^6^ DCs of each group in a 100 μL volume. During the first 5 days after DC transfer, animals of each group received a subcutaneous treatment (100 μL at the same site of DC inoculation) as follows: PBS, *A. sculptum* saliva (1:50 dilution) and PGE_2_ (100 nM). After 30 days of DC transference, the spleen was collected and the cells were restimulated with medium or HKRr for 72 h. The levels of IFN-γ **(A)** and IL-4 **(B)** were evaluated in cell-free supernatant of cultures by ELISA. The IFN-γ/IL-4 ratio is also presented **(C)**. Results are expressed as the mean ± SEM. ^*^p ≤ 0.05 vs. “DC + *R. rickettsii*/PBS” group.

When maintained in medium only, spleen cells of mice adoptively transferred with DCs incubated with *R. rickettsii* and submitted to different treatment protocols produced similar low basal levels of IFN-γ and IL-4 (Figures 6A,B). The *in vitro* stimulation of these cells with HKRr (recall response) induced a statistically significant increase of IFN-γ production in all three groups when compared to their respective controls, although no differences were observed among these groups (Figure 6A). The stimulation with HKRr also induced a slight increase in the IL-4 production by spleen cells of all groups receiving DCs incubated with *R. rickettsii*. In comparison with their respective controls, this increase only reached statistical significance in the group receiving DCs incubated with *R. rickettsii* and treated with PGE_2_; however, no differences were observed among the experimental groups (Figure 6B). In an attempt to estimate if the different treatments could induce any change in T cell polarization, the IFN-γ/IL-4 ratio was calculated for each group. Despite the increased polarization to the Th1 profile observed in cells stimulated *in vitro* with HKRr (over 200:1 ratio), no significant differences were observed among the three experimental groups (Figure 6C). Of note, qPCR analysis did not detect bacteria in spleen, suggesting that at this moment of the infection, no bacterial cell was present in mice spleens (data not shown).

**Figure 7:**
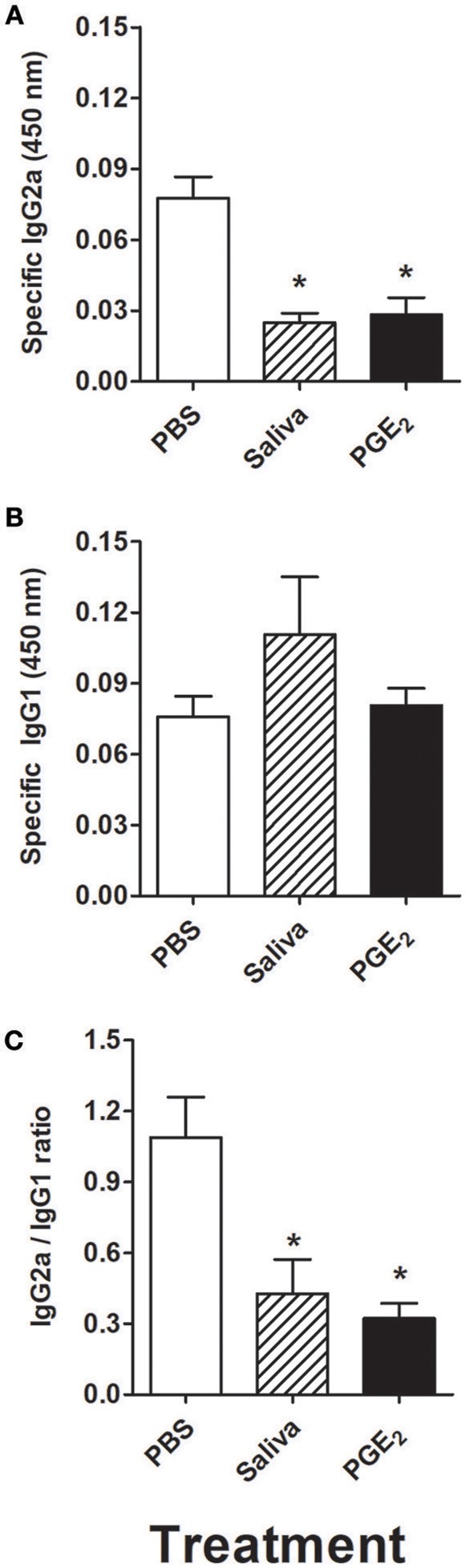
Adoptive transfer of murine DCs incubated with *R. rickettsii* and treated with *A. sculptum* saliva or PGE_2_ decreases specific IgG2a without changing IgG1 levels in serum. Bone marrow cells from C3H/HePas mice were differentiated into DCs, seeded at 10^6^ cells/well in 24-well plates and preincubated for 1 h with medium only, *A. sculptum* saliva collected by pilocarpine stimulation from female ticks fed on host for 7–9 days (1:50 dilution) or PGE_2_ (100 nM). Cells were incubated with *R. rickettsii* (10^7^ bacteria) during 18 h, collected and washed to remove unbound bacteria. Mice were injected subcutaneously with 10^6^ DCs of each group in a 100 μL volume. During the first 5 days after DC transfer, animals of each group received a subcutaneous treatment (100 μL at the same site of DC inoculation) as follows: PBS, *A. sculptum* saliva (1:50 dilution) and PGE_2_ (100 nM). After 30 days of DC transference, a blood aliquot was collected and the levels of *R. rickettsii*-specific IgG2a **(A)** and IgG1 **(B)** antibodies were evaluated in serum samples by ELISA. The IgG2a/IgG1 ratio is also presented **(C)**. Results are expressed as the mean ± SEM. ^*^p ≤ 0.05 vs. “DC + *R. rickettsii*/PBS” group.

The serum obtained from mice adoptively transferred with murine DCs incubated with *R. rickettsii* was evaluated for the presence of specific IgG2a and IgG1 antibodies. Figure 7A shows that specific IgG2a was significantly decreased in the serum of mice adoptively transferred with DCs incubated with *R. rickettsii* and treated with saliva or PGE_2_, when compared to the serum of mice treated with PBS (vehicle). On the other hand, specific IgG1 levels were similar among all groups (Figure 7B). The evaluation of IgG2a/IgG1 ratio revealed a negative balance toward IgG2a (below 0.5:1 ratio) in the groups that received DCs incubated with *R. rickettsii* followed by treatment with *A. sculptum* saliva or PGE_2_, when compared with the PBS-treated group (~1:1 ratio; Figure 7C).

## Discussion

Several DC populations are intimately associated to the skin immune system and present a number of biological, sometimes contradictory, specialized functions (18, 19, 59). In addition, in the last 40 years, a detailed interaction between DCs and tick saliva has been uncovered by both *in vivo* and *in vitro* studies (25–35). However, only two studies evaluated aspects related to the role of *A. sculptum* saliva in DCs up to date (34, 60), and none of them isolated or characterized the immunomodulatory molecule(s) responsible for the activities described.

In the present work, we demonstrated that *A. sculptum* saliva is able to decrease the production of inflammatory cytokines by LPS-stimulated DCs, regardless of the agonist employed for salivation (pilocarpine or dopamine). Reversed-phase UFLC fractionation showed that fractions eluting from 49 to 55% of ACN presented inhibitory activity on LPS-induced TNF-α production by DCs. The elution profile matches with PGE_2_ as demonstrated by previous studies (36, 40). In addition, an ELISA assay showed a direct proportional association between the potency of this activity and the PGE_2_ concentration in the fractions. However, a recent study on eicosanoid profiling of biological samples revealed that PGE_2_ and PGD_2_ cross-react in immunoenzymatic assays (50). In order to ascertain whether PGE_2_ really is the bioactive salivary molecule found in the present work (and perhaps all the other studies describing this lipid mediator in tick saliva), we employed a new high-resolution target mass spectrometry methodology recently described and validated to unambiguously characterize PGE_2_ as a major DC modulatory molecule in *A. sculptum* saliva. Although PGE_2_ and PGD_2_ are molecular isomers and present the same fragmentation profile and molecular mass characterization, both analytes can be separated by ultra-high-performance liquid chromatography system in a binary gradient (50) and, consequently, they can be identified and quantified as different lipid compounds in a complex biology mixture. Though a previous study identified the presence of PGE_2_ in *I. scapularis* saliva by a shotgun LC-MS approach (31), the method only detected the precursor ion in low resolution and this produced a bias on the presence of the target analyte considering the numbers of isomers in complex biological matrices. Herein, we used an advanced mass spectrometry assay (LC-TOF-MS/MS) in MRM^HR^ for target lipidomics, which identify the molecular mass of precursor ion and all the fragment ions in high-resolution with specific retention time in LC, that give us no doubt about the molecular identity of PGE_2_ in the *A. sculptum* saliva.

Some protein modulators of DC biology have been described in tick saliva of different species. The production of inflammatory cytokines by DCs, as well as the expression of DC activation surface markers, among other activities, are inhibited in the presence of salivary Salp15, sialostatin L1 and sialostatin L2 from *I. scapularis* (36–39), OmC2 from *O. moubata* (42, 43), Japanin from *R. appendiculatus* (41), and DsCystatin from *Dermacentor silvarum* (61). In most cases, the concentration of these molecules employed in these published studies were in the micromolar range, a concentration far superior to the amount of any protein naturally found in tick saliva. In this regard, even considering theoretical differences in the potency of the activity between native and the recombinant proteins, the only DC immunomodulatory molecules identified in tick saliva and confirmed in the salivary cocktail at pharmacological levels are PGE_2_ and adenosine, which are non-protein molecules (36, 40). Cytokine binding proteins are not likely to influence our findings because all of such molecules described so far were much larger than 3 kDa (62–64). Confirming this assumption, there was a lack of activity of the HMW fraction in LPS-stimulated DCs. In addition, no anti-TNF-α activity was found in saliva of *Amblyomma americanum*, suggesting that *Amblyomma* spp. may be devoid of this activity (65). On the other hand, considering that *R. rickettsii* is transmitted to the host since the first days of tick attachment and that PGE_2_ concentration in the saliva is relatively low during this initial period, the role of the other DC salivary modulators cannot be ruled out. Thus, the present study reinforces an important role of PGE_2_ on DC-*A. sculptum* saliva interactions, acting either additively or synergistically with other protein immunomodulators.

The SAT phenomenon has been reviewed in the context of tick saliva for many pathogens (3, 66). Nonetheless, few studies evaluated the SAT effect through the *Amblyomma*-*Rickettsia* association (67–69) and none of them specifically addressed the *R. rickettsii* interaction with *A. sculptum*. Experimental models for rickettsial infections that closely reproduce human disease are largely lacking, particularly for *R. rickettsii* (70, 71), even though a murine model using the C3H/HeN has been successfully used in the last few years to trial potential vaccine candidates to *R. rickettsii* (52–57, 72). Our attempts to use the C3H/HePas strain failed in generating a model of susceptibility to our *R. rickettsii* isolate (data not show), although this murine strain is known to be closely related to the C3H/HeN.

Another aspect not appropriately covered by the literature is that few reports characterized the infection of murine DCs by *Rickettsia* spp. (58, 73–75). Here we showed, for the first time that *R. rickettsii* is able to infect murine DCs and induce a proinflammatory cytokine profile by these cells. This same proinflammatory phenotype was observed in human DCs incubated with *R. rickettsii*. We also demonstrated that *A. sculptum* saliva and PGE_2_ inhibit this profile in both murine and human DCs, reinforcing their modulatory activity on a rickettsial-DC context. Of note, the *R. rickettsii*-induced expression of CD40, CD80, and CD86 presented a particular profile for murine and human DCs and was not consistently downmodulated by the presence of saliva or PGE_2_. This aspect is not covered by the literature and more studies are needed to establish the differences and similarities in the DC responses to rickettsial infection between the two species.

Considering the role of DC as the most important antigen-presenting cell in the skin and other organs (23), the modulation of DC phenotype by tick saliva may have an impact on the *in vivo* immunity to *R. rickettsii*. Thus, we assessed whether the adoptive transfer of murine DCs incubated with *R. rickettsii* and treated with *A. sculptum* saliva or PGE_2_ would induce changes in humoral and cellular immune responses against *R. rickettsii* in C3H/HePas mice. The recall responses showed the production of high levels of IFN-γ and a marginal increase of IL-4 by spleen cells of DC-transferred mice stimulated with HKRr when compared to cells maintained in medium. In fact, IFN-γ is associated to host defenses against rickettsial infections (76, 77) and the IFN-γ-related response is reportedly associated with resistance to the infection in vaccination studies of mice against *R. rickettsii* (53–55). More recently, protective peptides derived from *R. rickettsii* antigens were shown to trigger a Th1 dominant response associated with IFN-γ production (56). On the other hand, tick infestation is known to increase the production of Th2 cytokines while inhibiting Th1 cytokines in mice (78, 79), but this polarization seems to be more effective in susceptible than in resistant mice (80), and under natural infection rather than through syringe inoculation (81). In addition, either DCs directly incubated with tick saliva *in vitro* or DCs isolated from mice treated with tick saliva induced a decrease of IFN-γ and stimulation of IL-4 production by CD4^+^ T cells (32). In the present study however, the treatment of DCs with *A. sculptum* saliva or PGE_2_ prior to incubation with *R. rickettsii* was not able to alter the IFN-γ/IL-4 ratio systemically developed by the mice injected with these cells.

Regarding the humoral immunity, *R. rickettsii*-specific IgG2a levels were diminished in the serum of mice receiving DCs incubated with the bacteria and treated with *A. sculptum* saliva or PGE_2_ while *R. rickettsii*-specific IgG1 levels remained unaltered. The role of IL-4 (originally described as a B cell stimulatory factor) in the B cell survival and switch to IgG1 was established more than 30 years ago (82, 83) and may help to explain the persistence of this isotype observed in the presence of saliva and PGE_2_. Earlier studies characterized murine monoclonal antibodies with protective activity against *R. rickettsii* and showed that most of them were of IgG2a isotype and none were of IgG1 isotype (84, 85). In addition, the protective properties of IgG2a antibodies against *R. rickettsii* infection *in vivo*, acting in synergy to other IFN-γ-dependent mechanisms, are supported by some studies (53–55). Furthermore, because mice were also treated with either saliva or PGE_2_ after DC transfer, a direct effect of the treatment on antibody class switch cannot be ruled out, as extensively demonstrated by many studies on B cells. For example, PGE_2_ was shown to promote the differentiation of B lymphocytes into IgG2a- (86), IgG1- and IgE-secreting cells (87–89), while IgM and IgG3 were inhibited in the same conditions (87). The basis of these differences may reside in the differentiation stage of B lymphocytes (e.g., resting *vs*. activated), the stimuli present in the environment (e.g., cytokines, pathogens or microbial products), the T helper population interacting with B cells and, ultimately, the set of PGE_2_ receptors expressed by these cells during a given immune response. In addition, there is an interesting hypothesis recently raised in that PGE_2_ from tick saliva would be the mediator responsible for allergy to red meat found in some individuals exposed to ticks. According to the authors, salivary PGE_2_ would trigger antibody class switch in type-A and -O individuals to induce anti-α-Gal IgE antibodies that break oral tolerance to food allergens (90). In line with this conjecture, specific IgG2a/IgG1 ratio was decreased following treatment with either *A. sculptum* saliva or PGE_2_, but whether these humoral changes are associated with increased susceptibility to *R. rickettsii* still remains to be determined.

In brief, the present work reinforces the anti-inflammatory role of tick saliva on murine and human DCs and shows that *A. sculptum* produces pharmacological levels of PGE_2_ in its salivary secretion. A new and robust mass spectrometry approach confirmed that tick saliva does not contain PGD_2_, which usually cross react with PGE_2_ in immunoenzymatic assays. Finally, we evidenced for the first time, the murine DC-*R. rickettsii* interactions and the ability of PGE_2_ to drive DC-dependent humoral changes to a rickettsial infection *in vivo*.

## Author Contributions

EE, BB, CLS, and AS-N designed the experiments. EE, AR-H, RT, FC, and AS-N generated biological samples. EE, BB, AP, AC, PW, CAS, and AS-N performed the experiments. EE, BB, AP, AC, CLS, LF, AF, CAS, and AS-N analyzed data. EE, BB, and AS-N performed statistic data analysis. PW, ML, PS, CLS, LF, AF, CAS, AS-N contributed reagents, materials, and analysis tools. EE, BB, AC, PW, AF, CAS, and AS-N wrote the paper. All authors read and approved the final manuscript.

### Conflict of Interest Statement

The authors declare that the research was conducted in the absence of any commercial or financial relationships that could be construed as a potential conflict of interest.
