# Supplementary figures and images for: Amblyomma sculptum Salivary PGE2 Modulates the Dendritic Cell-Rickettsia rickettsii Interactions in vitro and in vivo
Source: Front Immunol. 2019 Feb 4;10:118. doi: 10.3389/fimmu.2019.00118 (PMC6369204; doi:10.3389/fimmu.2019.00118)

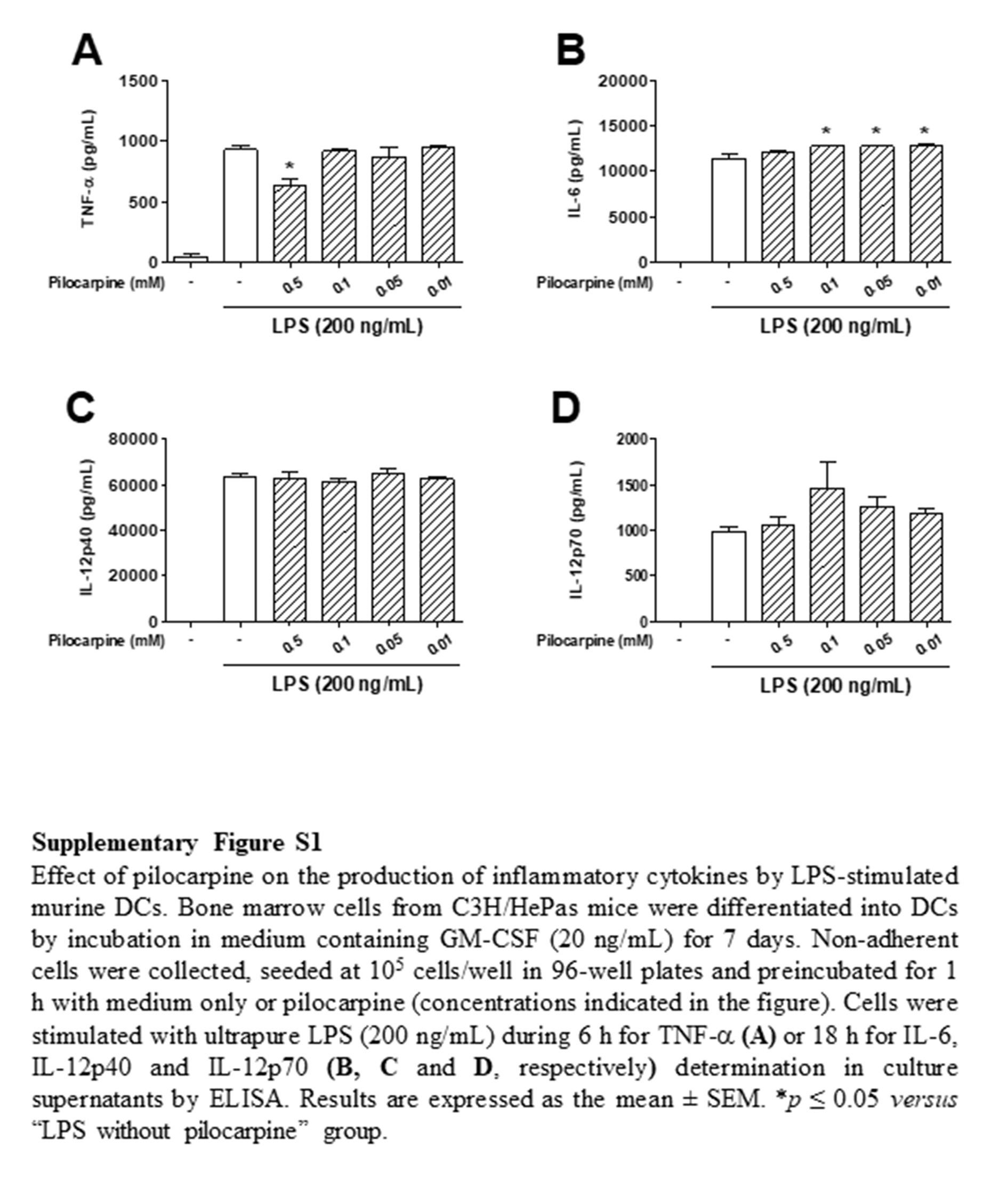

Supplement: Supplementary file 1 [file Image_1.TIF]

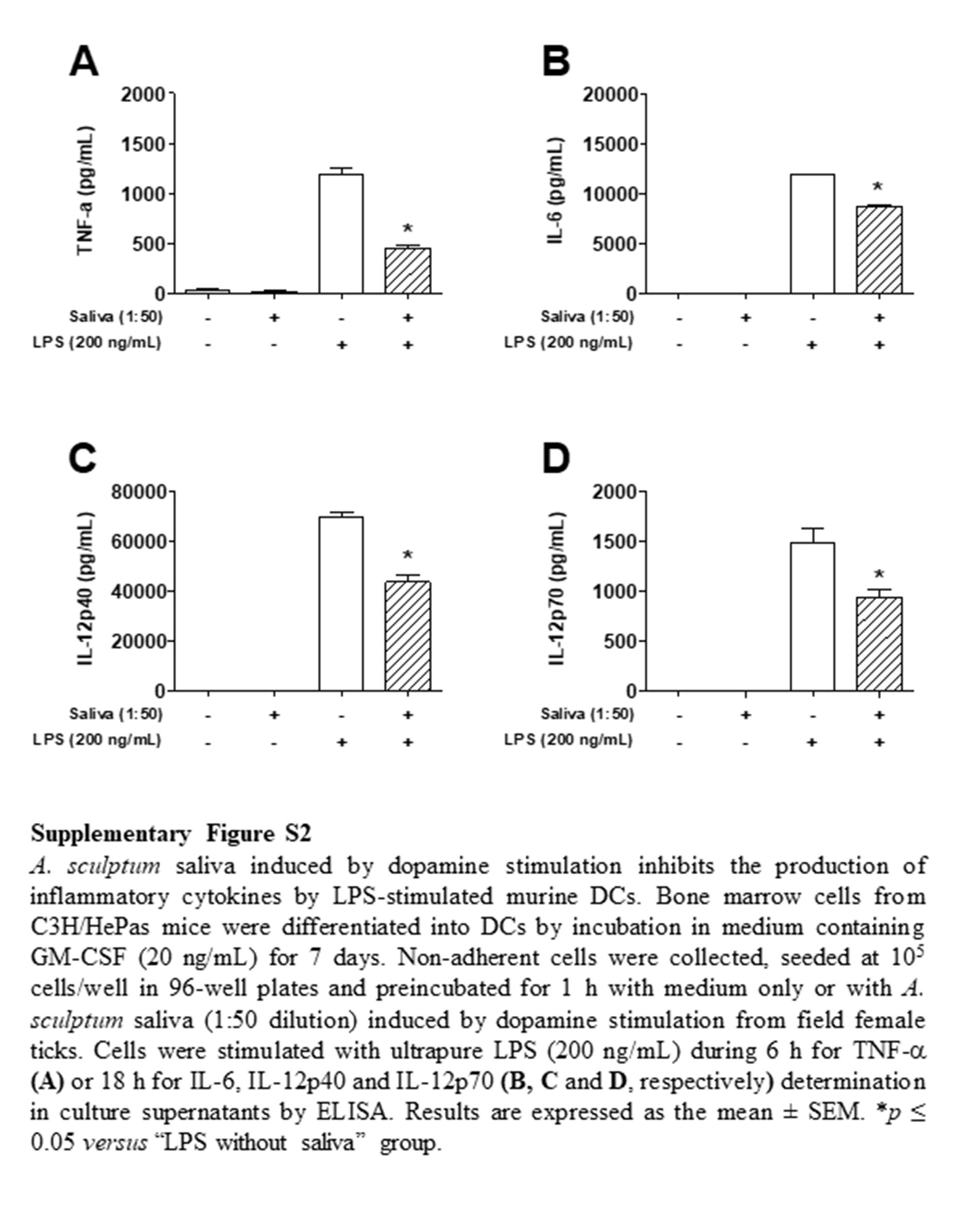

Supplement: Supplementary file 2 [file Image_2.TIF]

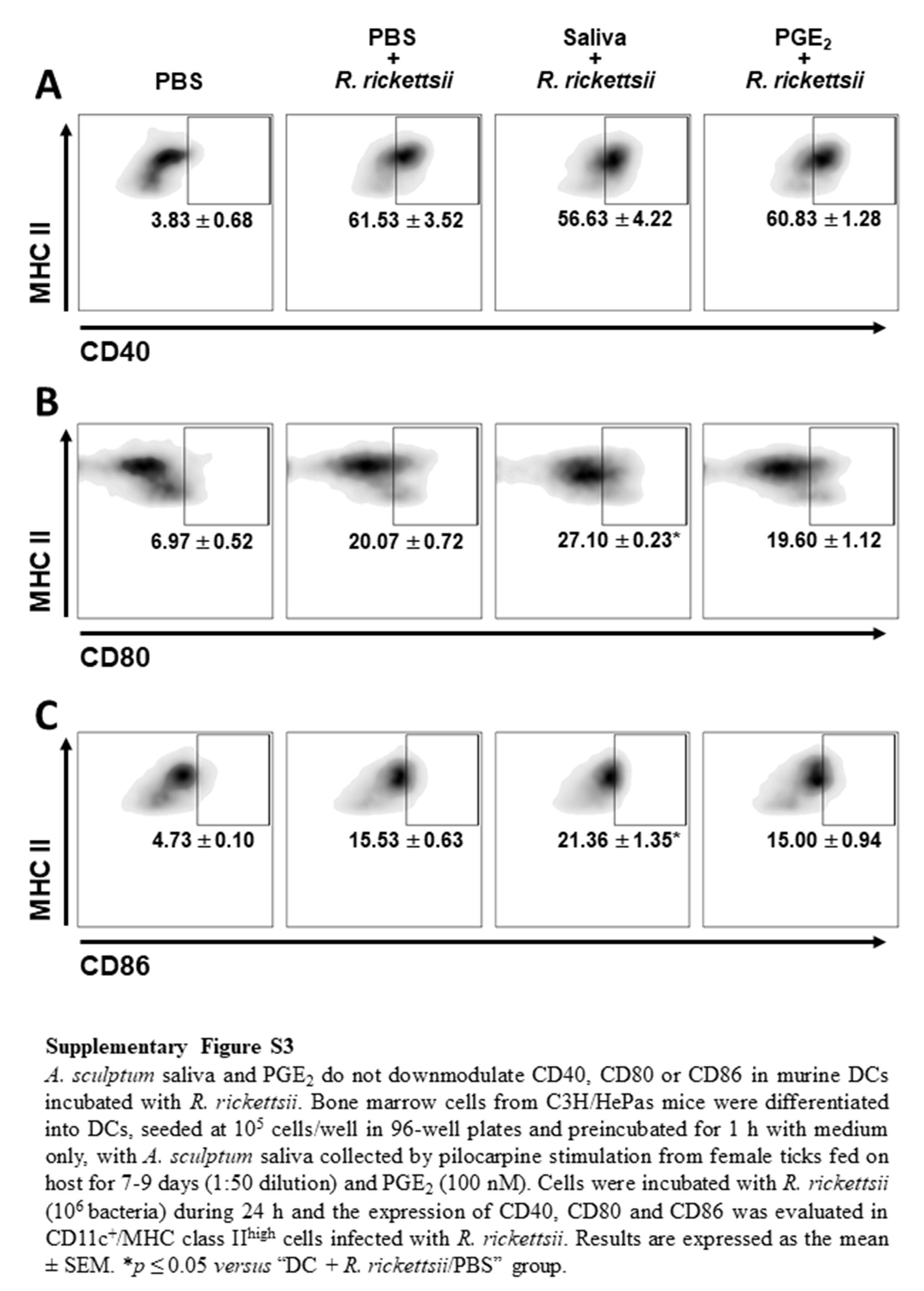

Supplement: Supplementary file 3 [file Image_3.TIF]

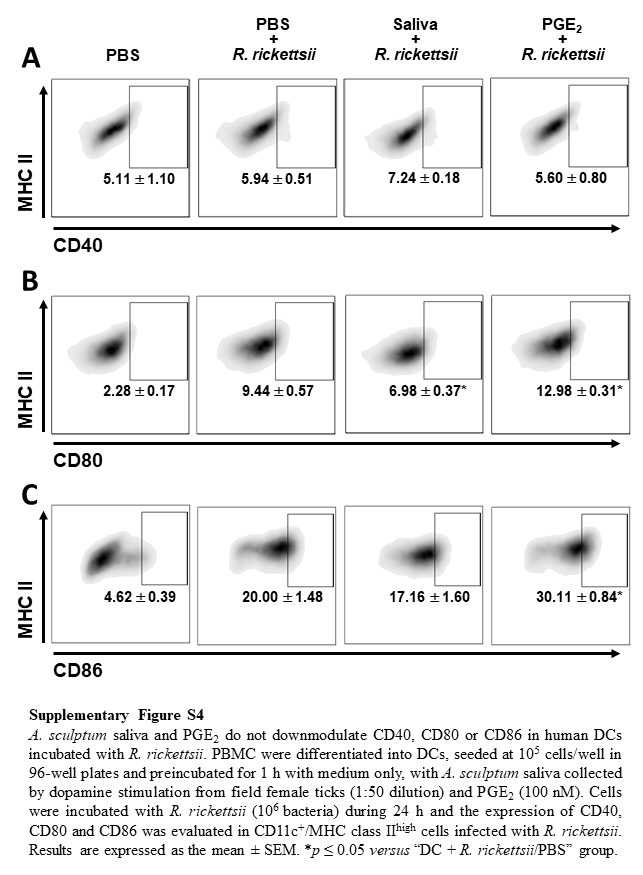

Supplement: Supplementary file 4 [file Image_4.TIF]
